# A New Approach to Evaluate Neuromuscular Fatigue of Extensor Elbow Muscles

**DOI:** 10.3389/fphys.2020.553296

**Published:** 2020-09-23

**Authors:** Matheus Silva Norberto, Tarine Botta de Arruda, Marcelo Papoti

**Affiliations:** ^1^Postgraduate Program in Health Sciences Applied to the Locomotor System, Ribeirão Preto Medical School, University of São Paulo, Ribeirão Preto, Brazil; ^2^School of Physical Education and Sport of Ribeirão Preto, University of São Paulo, Ribeirão Preto, Brazil

**Keywords:** neuromuscular fatigue, twitch interpolation technique, elbow extension, sports science, triceps brachii, percutaneous superimposed electrical stimulation technique

## Abstract

Neuromuscular fatigue evaluation is widely performed on different muscles through the conventional protocol using maximum voluntary contraction (MVC) with electrical stimuli in the analyzed muscle. In an attempt to use this protocol on elbow extensor musculature, previous studies and pilot studies showed co-contraction effects from antagonist musculature during muscular stimulations. The aim of this study was to propose a new neuromuscular fatigue protocol evaluation on elbow extensor musculature. Twenty participants preformed exercises to induce central (CenFat) and peripheral fatigue (PerFat). Neuromuscular fatigue was evaluated on knee extensor muscles by a conventional protocol that provides Twitch Superimposed (TS_K_) and Twitch Potentiated (TP_K_), central and peripheral parameters respectively. For elbow extensor muscles, the protocol used sustained submaximal contraction at 10, 20, 30, 40, and 50% of MVC. The neuromuscular fatigue in upper limbs was identified by Twitch Potentiated (TP_E_) and multiple Twitch Superimposed (TS_E_) parameters. Using the relationship between MVC (%) and evoked force, the proposed protocol used several TS_E_ to provide slope, y-intercept and *R*^2^. It is proposed that slope, *R*^2^, and y-intercept change may indicate peripheral fatigue and the identified relationship between y-intercept and *R*^2^ may indicate central fatigue or both peripheral and central fatigue. The results were compared using the non-parametric analyzes of Friedmann and Wilcoxon and their possible correlations were verified by the Spearmann test (significance level set at *p* < 0.05). After PerFat a decrease in TP_E_ (57.1%, *p* < 0.001) was found but not in any TS_E_, indicating only peripheral fatigue in upper limbs. After CenFat a decrease in TP_E_ (21.4%, *p*: 0.008) and TP_K_ (20.9%, *p* < 0.001) were found but not in TS_K_, indicating peripheral fatigue in upper and lower limbs but not central fatigue. A non-significant increase of 15.3% after CenFat and a statistical reduction (80.1%, *p*: 0.001) after PerFat were found by slope. Despite *R*^2^ showing differences after both exercises (*p* < 0.05), it showed a recovery behavior after CenFat (*p*: 0.016). Although PerFat provided only peripheral fatigue, CenFat did not provide central fatigue. Considering the procedural limitations of CenFat, parameters resulting from the proposed protocol are sensitive to neuromuscular alteration, however, further studies are required.

## Introduction

The conventional neuromuscular fatigue evaluation proposed by Merton (1954) comprise the measurement of maximum voluntary contraction (MVC) with local muscular stimulation. The difference between MVC and the force evoked during electrical stimulation is denominated “Twitch Superimposed” (TS) (Gandevia, 2001). Increases in this parameter are associated with central fatigue (Allen et al., 1995; Gandevia, 2001). In order to measure the peripherical pathway, after TS induction, another muscular stimulation is applied to the relaxed muscle. In this case, the evoked force is “Twitch Potentiated” (TP) (Gandevia, 2001). This protocol is also able to provide information on voluntary activation, thus being an important parameter in sports and the clinical-hospital environment (Shield and Zhou, 2004; Paillard et al., 2005).

Despite this method’s effectiveness as a procedure for musculature such as the quadriceps femoris (Bigland-Ritchie et al., 1978; Strojnik, 1998; Becker and Awiszus, 2001; Place et al., 2007; Martin et al., 2010; Girard et al., 2013; Milioni et al., 2016), studies that used this protocol in the elbow extensor found limitations and difficulties for interpretation of their results (Lloyd et al., 1991; Allen et al., 1994; Dowling et al., 1994), suggesting that this strategy may have its validity compromised for this region.

A possible frailty in triceps brachii in tasks involving maximum contractions was suggested, being susceptible to present synergistic muscle inhibitory effects (co-contraction phenomenon) such as the biceps brachii (Awiszus et al., 1997) and the influence of neural reflex (collision between orthodromic and antidromic potentials capable of decreasing the response to stimulation) (Herbert and Gandevia, 1999; Shield and Zhou, 2004). There is evidence that the triceps brachii is also prone to the development of central fatigue (Bilodeau et al., 2001; Bilodeau, 2006), peripheral fatigue (Meszaros et al., 2010), and post-activation potentiation (Hamada et al., 2000) depending on the voluntary contraction duration performed by this muscular region.

Regarding neuromuscular fatigue analysis for elbow extensor musculature (Millet and Lepers, 2004), stimulation in submaximal contractions may be a good strategy since it is effective to measure fatigue but is insufficient to generate fatigue or potentiation (Hamada et al., 2000; Bilodeau, 2006; Meszaros et al., 2010). Furthermore, stimulation in submaximal contractions have been successfully used for voluntary activation measurement in special populations, patients with limited conditions (Rutherford et al., 1986) and/or individuals with specific muscular groups that present motor limitations as an impairment to muscular length (Cheng and Rice, 2010).

Neuromuscular fatigue evaluation through muscle stimulation during a maintained submaximal contraction (30% of MVC) has already been evidenced in the lower limbs (Bigland-Ritchie et al., 1986). Moreover, previous studies used stimulation at different submaximal voluntary contraction rates to estimate TP on triceps brachii (Cheng and Rice, 2010) and quadriceps femoris (Becker and Awiszus, 2001; Temesi et al., 2014) through extrapolation of the results obtained by linear regression (relation between the force evoked during stimulation in different submaximal contractions). In addition, extrapolation has also been used to determine values of evoked force in high voluntary contractions using curvilinear methods in lower limb musculature (Behm et al., 1996).

It is important to note that Merton (1954) was the first researcher to suggest the linear method for extrapolating values of force evoked in submaximal contractions to estimate values referring to higher contractions. Years later, studies also tested curvilinear methods (Dowling et al., 1994) and linear (De Serres and Enoka, 1998) to estimate TS parameter on MVC and, consequently, calculate the voluntary activation for the elbow flexor musculature. Futhermore, the linearity of the force evoked in the brachial biceps by transcranial stimulation also used to extrapolate values of 100, 75, and 50% of the MVC to establish the value of force evoked at 0% (Todd et al., 2004; Cadigan et al., 2017).

The linear method (linear regression) made by several TS, resulting from stimulation at different submaximal contraction rates, may be a valid approach to compare the neuromuscular status (differentiate and/or quantify the type of fatigue), providing parameters capable of identifying central and/or peripheral fatigue. It is possible that this mathematical strategy is sensitive, since a linear relationship between the evoked force and the submaximal contraction rate has already been verified (Bigland-Ritchie et al., 1986; De Serres and Enoka, 1998; Perumal et al., 2010).

Analysis that employs linear regression presupposes that the line extremities (arising from the relations of force evoked during the submaximal contractions stimulation) are directly regulated by parameters already discussed in the literature, such as pulses applied with the relaxed musculature (TP) (representing one extremity of the line) and the use of 100% TS of MVC (representing another extremity of the line) (Allen et al., 1995; Gandevia, 2001).

In the abstinence of detailed studies with triceps brachii, we consider that for the biceps brachii, both curvilinear and linear methods are valid and similar to extrapolate the parameters (De Serres and Enoka, 1998). In this case, the multiple TS parameters, resulting from stimulation in different submaximal voluntary contractions, besides providing more points in the linear method, may have utility to measure the neuromuscular imbalance after a fatigue task with less risk to fatigue establishment (Place and Millet, 2020). The hypothesis regarding this strategy is based on the following arguments: if stress in the peripheral pathway reduces TP (Gandevia, 2001), a parameter that is an integral part of linear regression (with other TS in submaximal contraction values), theoretically it will reduce the angular coefficient (slope) and linear coefficient (y-intercept). Whereas this stress would not result in significant changes to the other line extremity, the dispersion of the points on the line would be compromised, indirectly reducing the determination coefficient (*R*^2^).

Although slope and y-intercept are also susceptible to alterations when exposed to simultaneous central and peripheral fatigue, the central pathway stress would provide a brief increase in extremity line parameters relative to TS applied to 100% of MVC (Allen et al., 1995), making the dispersion of the linear method more grouped, a scenario that would result in a higher *R*^2^ than in the situation where the participant would be exposed only to peripheral pathway stress. In the latter case, exposure only to central pathway stress compromises both slope and *R*^2^, considering that the y-intercept remains the same as a function of the TP value that remains unchanged (Gandevia, 2001).

Finally, the use of y-intercept and the angular coefficient, assuming that the relationship between electrical stimulations in different submaximal contractions and evoked force satisfactorily represents the biological phenomena of interest, can be a viable alternative to neuromuscular fatigue evaluation in muscles that can be affected by the “co-contraction phenomenon” or prone to fatigue and potentiation as triceps brachii (Hamada et al., 2000; Bilodeau, 2006; Meszaros et al., 2010). Considering the understanding that these responses are inherent to the evaluated region and that trying to circumvent them may limit the interpretation of the real neuromuscular state of the investigated portion, and that at high intensity of electrostimulation in the triceps brachii muscle the phenomenon of co-contraction is inevitable, we aim at a protocol that considers the nature of the musculature (Place and Millet, 2020) and still provides information about the neuromuscular state. Thus, the purpose of this study was to verify the effectiveness of a protocol applied on elbow extensor muscle to measure neuromuscular fatigue from the evoked force using maintained submaximal contractions.

In order to guarantee the validation of the new proposal for neuromuscular assessment in elbow extensor musculature, neuromuscular fatigue evaluation in the lower limbs was used, due to the fact of having proven scientific validity (Allen et al., 1995; Becker and Awiszus, 2001; Gandevia, 2001). In addition to the neuromuscular evaluation for lower limbs being already valid, this appropriateness was chosen with the objective of using lower limbs as a counterpart of the central fatigue provider.

## Materials and Methods

### Participants

Twenty subjects who exercise regularly (performed low or moderate intensity physical exercises two to five times a week) participated in the study with a mean age, body mass and height of 25.1 ± 6.8 years, 74.9 ± 11.4 kg, and 174.8 ± 9.7 cm respectively. The participants were: five females (18–25 years; 161–174 cm; 58–75 kg) and 15 males (19–43 years; 170–191 cm; 62–90 kg). After divulgation of the research and contact of interested participants, individual contact was made with each participant to explain the risks, procedures used, and study objectives. The participants were asked not to train or use any ergogenic, energy, or alcoholic resources in the 48 h prior to the evaluations. Participants were only included in the study after signing the consent form. All experiments were previously approved by the Ethics Committee (Process number: 97168618.4.0000.5659) and conducted in accordance with the Declaration of Helsinki.

### Design and Procedures

After three familiarization sessions, the volunteers made two visits to the laboratory to realize the principal focus of the present study: validation of the proposed evaluation protocol for elbow extensor musculature. In the two visits the participants made the same experimental design that included: pre-evaluation, fatigue induction by an exercise, and post-evaluation. However, each of these visits was covered with a different exercise (induce central or peripheral fatigue) with randomly established sessions. Both environments had a controlled temperature of 71.6°F.

Neuromuscular evaluation in lower limbs, due to the fact of having proven scientific validity (Allen et al., 1995; Becker and Awiszus, 2001; Gandevia, 2001), aimed to establish support information for the proposed protocol. Therefore, the present study also included neuromuscular evaluation on knee extension musculature (valid protocol) in the same evaluation chair (built specifically for neuromuscular fatigue evaluation for both portions). It is important to highlight that once central fatigue is detected by the lower limbs, it is a state of fatigue that affects the whole body (Bangsbo et al., 1996; Gandevia, 2001; Johnson et al., 2015). That is, the valid protocol for knee extensor musculature served as a basis for validation of the proposed evaluation protocol. Respecting the existing knowledge about neuromuscular fatigue (Gandevia, 2001), this study provides a different approach to the treatment of results through linear regression (covered in the following topics).

**FIGURE 1:**
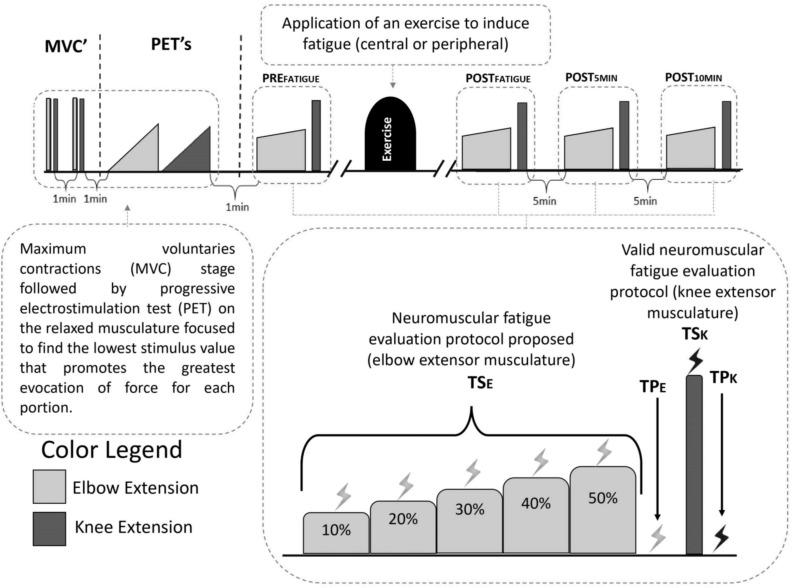
Experimental design adopted in this study. Measurements made on the elbow extensor musculature are illustrated in light gray and measurements made on the knee extensor musculature are illustrated in dark gray. The experimental steps included: assessment of maximum voluntary contraction (MVC), progressive test of electrostimulation (PET) to establish the best value for stimulation and pre and post-exercise evaluations. The evaluations included: for elbow extensor musculature, submaximal contractions maintained in 10, 20, 30, 40, and 50% of the MVC on elbow extensor musculature with stimulus applied to each maintained force (TS_E_) and another stimulus applied with the relaxed musculature at the end of the submaximal task (TP_E_); for knee extensor musculature, a maximum contraction accompanied by a stimulation (TS_K_) followed by another stimulation applied to the relaxed musculature (TP_K_).

The experimental design involved five steps, which were repeated identically on the two different days in which the participants performed the exercise to promote fatigue: (1) The maximal voluntary isometric contraction of elbow (MVC_E_) and knee (MVC_K_) was determined; (2) The progressive electrostimulation test (PET) were performed for the elbow extensor and knee flexor musculature to provide the lowest stimulation intensity that promotes the highest rate of evoked force (best stimulation rate). This was performed in the moment before the fatigue induction exercise (PRE_FATIGUE_); (3) The application of the valid neuromuscular fatigue evaluation protocol for knee extension musculature (Merton, 1954; Allen et al., 1995) and the proposed protocol for elbow extensor musculature. Both neuromuscular evaluations were performed against a load cell to measure the force response (explained in detail in the following topics); (4) The participants were submitted to pre-established exercises to induce predominant neuromuscular fatigue with a central (Linnamo et al., 1997; Peltonen et al., 2013) or peripheral (Dundon et al., 2008) feature; (5) The neuromuscular fatigue evaluation protocols were performed again (identical to the third step) exactly at the end of the exercise (POST_FATIGUE_) and during the recovery (POST_5__MIN_ and POST_10__MIN_ respectively) in both muscular regions (Figure 1).

### Measures: Stimulation and Torque

The optimized intensity of the electrostimulation was determined individually for each participant (both for upper and lower limbs) using the PET (in the first step). Electrical stimulation was performed through an electrostimulator prototype developed for this purpose with a 200 V peak-to-peak capacity (Bioestimulador, Insight^®^, Ribeirão Preto, Brazil). Elbow and knee extension were performed against different load cells (CSR-1T, MK Control^®^, São Paulo, Brazil) and data acquisition was made with a Labview 2015 environment (National Instruments^®^) with 1000 Hz acquisition frequency.

For the muscular belly stimulation, two round electrodes (3 cm diameter) were used in the triceps brachii (ValuTrode^®^ CF3200 Self-Adhesive Electrodes) (Lampropoulou et al., 2012) and two 5 cm × 9 cm rectangular electrodes were used in the quadriceps femoris (adhesive electrodes CF5090 ValuTrode^®^). For lower limbs stimulation electrodes were positioned at the upper limit (cathode) and lower limit (anode) of the *vastus lateralis* and for upper limbs stimulation were positioned on the triceps long head (cathode) and distal tendon (anode). It should be noted that the electrical pulses applied in the musculature were “doublets.” Doublets guarantee force evocation during muscle stimulation (Behm et al., 1996; Gandevia, 2001) and are made by “duplicate stimulations” that have a duration of 1 ms and the interval between each stimulation (referring to duplicate) of 10 ms.

A specific chair for neuromuscular evaluation was used for force measurement, and consequently neuromuscular evaluation, so that the participant remained with the trunk supported by the chair with the arm relaxed and parallel to the trunk, while the elbow was maintained in 90° flexion. The load cell attached to a sliding block had an adjustable strap that was attached to the participant’s wrist (dominant arm). To perform submaximal contractions, the load cell was adapted into a routine where the participant had access to its simultaneous strength by visual access to a force target through a monitor. The second load cell, for knee extension force evaluation, was attached to the chair and fastened to the participant’s ankle (dominant foot) while their leg was kept relaxed with 90° of knee flexion, without visual force information.

The order of evaluation was the upper limbs and lower limbs respectively to ensure the quality of neuromuscular responses to the proposed protocol. It should be noted that in less than a minute after the end of the exercises, the entire neuromuscular evaluation protocol (including upper and lower limbs) had already been performed, preserving the neuromuscular responses with the best quality possible (Gandevia, 2001; Gandevia et al., 2013).

### Neuromuscular Fatigue Protocol Evaluation Proposed

The elbow extension protocol involved an evaluation where the participant maintained sequentially five submaximal intensities of MVC (10, 20, 30, 40, and 50%) for 5 s each. During each submaximal contraction, stimulations were applied and the evoked force during the stimulation was determined by the difference between the submaximal contraction force and the submaximal contraction rate maintained (TS_E_). At the end of the submaximal task another stimulation was applied on relaxed muscle and the difference between the evoked force and weight value of the relaxed musculature was considered as TP_E_.

Considering that the most common evaluation model uses stimulation in maximal contractions and that this model does not work well with the elbow extensor musculature due to the fact of stimulation superimposed on maximal contractions not exhibiting evoked force impairment (Burke and Gandevia, 1998; Cheng and Rice, 2010; Temesi et al., 2014), the use of submaximal contractions, and later, the use of the relationship between the evoked force and the maintained sub-maximum rate, allows to identify products of linear regression (slope, y-intercept, and *R*^2^).

This hypothesis is based on the basic principles of neuromuscular analysis established by Gandevia (2001) that can support: peripheral and central fatigue that may show a reduction in y-intercept and slope with maintenance of *R*^2^ (considerable reduction in TP and adjustment by TS in higher submaximal contractions); peripheral fatigue may show a reduction in y-intercept, slope, and *R*^2^ (considerable reduction in TP and discreet adjustment by TS in higher submaximal contractions) and; central fatigue may show maintenance of y-intercept with reduction of slope and *R*^2^ (maintenance of TP and adjustment by TS in higher submaximal contractions).

### Valid Neuromuscular Fatigue Protocol Evaluation for Knee Extensor Musculature

The procedure to identify neuromuscular fatigue for knee extension musculature was composed of a 5 s MVC with a stimulation on the third second (TS_K_) and a stimulation with relaxed muscle 3 s after MVC (TP_K_). This procedure was able to identify the peak force and, through the TP_K_ and TS_K_, it was possible to calculate the voluntary activation (VA_K_ (%) = [1 - (TS_K_/TP_K_)] ^∗^ 100) (Allen et al., 1995).

### Workout to Induce Central and Peripheral Fatigue

To induce peripheral fatigue in the elbow extensor musculature, a specific exercise for the region was chosen, while in view of generating only central fatigue, an exercise involving only lower limbs was preferred. Once the central fatigue is installed via the lower limbs, it is hypothesized that the upper limbs will only be able to change their parameters related to the central pathway (Bangsbo et al., 1996; Johnson et al., 2015).

The peripheral fatigue induction exercise (PerFat) consisted of the halter elevation exercise (made behind the head), which was performed in the neuromuscular fatigue evaluation chair. The halter starts from an initial position with 180° shoulder flexion and 35° elbow flexion, ending the movement with 180° elbow extension, giving a 135° movement degree. As in the original exercise the volunteers only performed the concentric movement (Dundon et al., 2008). As used by the study authors (Dundon et al., 2008), this exercise uses the equivalent of 30% of previous MVC_E_ load (made in the pre-evaluation stage) in the halter exercise. Each series was composed of 10 repetitions with a 20 s interval between each series (Dundon et al., 2008). When difficulty in performing the exercise was observed, the participant received minimum help to finish the series. From this moment, all subsequent intervals were accompanied by a quick force evaluation (3 s MVC). When a 30% reduction in MVC_E_ value was observed, the exercise was interrupted and the post-neuromuscular fatigue evaluation started (the fifth step of the evaluation protocol) (Dundon et al., 2008).

The central fatigue induction exercise (CenFat) was the leg extension exercise (Linnamo et al., 1997) in a maximal strength routine (Peltonen et al., 2013) and consisted of 15 sets of one repetition with maximum load in the leg-press 45° apparatus (FlexFitness Equipaments^®^). After the warming up stage (20–30 repetitions with subjectively low load), the participant was asked to establish a load for which they could perform a maximum of 10 repetitions. The load and the number of repetitions to perform this task were applied to Brzycki (1993) equation, a valid method to establish the maximum load value in leg-extension exercises (Menêses et al., 2013). The load could increase according to the participant’s feedback and could reduce in case of inability to perform the exercise.

**FIGURE 2:**
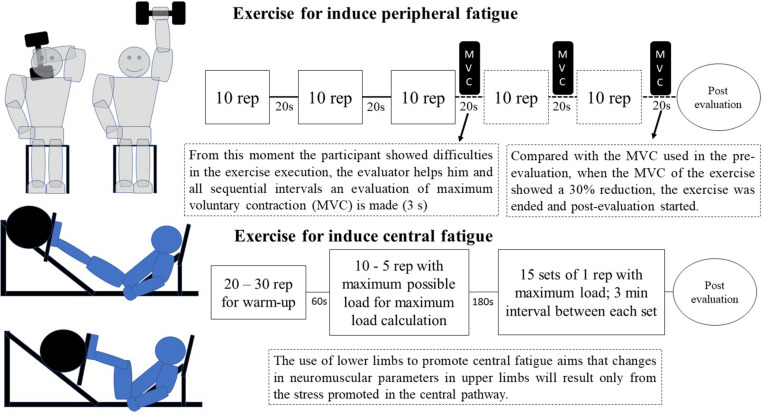
Model of exercises used to induce central fatigue and peripheral fatigue. The exercises were made on different days.

The choice of central fatigue inducing-exercise was made considering that central fatigue is more quickly established when there is greater muscle involvement in the effort (Gandevia, 2001), while the training composition was chosen because maximal strength is an excellent proposal for central fatigue compared to explosive strength training or hypertrophy (Peltonen et al., 2013) (Figure 2).

### Statistical Analysis

Using software G^∗^Power (version 3.1.1.9 – Universität Kiel, Germany), it was possible to identify that 19 participants were necessary for the present study to obtain a significant statistical power (sample power of 0.80 and alpha of 0.05). Data were processed using JASP^®^ (Version 0.12.2). Due to the lack of normality and sphericity, the data were treated in a non-parametric manner. In order to investigate the difference between situations, moments, and multiple pulses, Friedmann’s variance ranking analysis and a Wilcoxon classification test were used. The Wilcoxon classification test was also used for the differences between the two exercises’ parameter results. A *p* < 0.05 significance level was considered for all results. Effect size is given by the matched Rank-Biserial Correlation.

The reliability of the data (comparison between different days) was also tested with intraclass correlation coefficient (ICC), typical error (TE), coefficient of variation (CV%), standard error of the measure (SEM), and minimal difference needed to be considered real (MD) (Hopkins, 2000; Weir, 2005). The TE corresponded to the standard deviation of the individual differences between test and retest situations divided by 1.42 (Hopkins, 2000). The CV% was assumed as the ratio between TE and the mean of all observed values. SEM was calculated by the product between the standard deviation of the differences (difference between days) and the root of 1 minus ICC (Weir, 2005). MD was calculated by the product between SEM and 2.77 (Weir, 2005). These procedures to test reliability were proposed by Hopkins (2000) and Weir (2005). ICC was considered poor (<0.5), moderate (0.5–0.75), good (0.75–0.9), or excellent (>0.9) (Koo and Li, 2016).

## Results

### Comparison Between the Moments Before and After Exercise

The PerFat exercise promotes statistical reduction on MVC_K_ between PRE_FATIGUE_ and the two last measurements (effect size: 0.699 and 0.804 for POST_5__MIN_ and POST_10__MIN_ respectively; *p* < 0.05). After CenFat, statistical differences were found for TP_K_ between the PRE_FATIGUE_, POST_FATIGUE_ and both recovery moments (effect size: 0.929, 0.919, and 0.867, respectively; *p* < 0.01). CenFat also shows statistical differences for MVC_K_ between the PRE_FATIGUE_, POST_FATIGUE_ and both recovery moments (effect size: 0.863, 0.968, and 0.999, respectively; *p* < 0.01) (Table 1).

**TABLE 1:** Differences between neuromuscular fatigue evaluation moments. Parameters obtained through muscle contraction and stimulation performed on knee extensor muscles.

**Exercise**	**Mom.**	**TP_*K*_ (N)**	**TS_*K*_ (N)**	**VA_*K*_ (%)**	**MVC_*K*_ (N)**
PerFat	PRE_*FATIGUE*_	254.4 ± 63.6	55.5 ± 38.7	81.93 ± 9.3	597.2 ± 104.9
	POST_*FATIGUE*_	262.6 ± 65.6	62.6 ± 50.6	81.17 ± 16.5	562.0 ± 139.7
	POST_5MIN_	253.1 ± 65.9	59.9 ± 40.5	80.85 ± 10.7	543.6 ± 119.5*
	POST_10MIN_	253.7 ± 65.0	63.4 ± 48.5	80.20 ± 11.2	538.5 ± 127.0*
CenFat	PRE_*FATIGUE*_	262.3 ± 58.1	62.9 ± 50.6	81.48 ± 11.9	546.0 ± 123.3
	POST_*FATIGUE*_	206.7 ± 44.9^$^	48.5 ± 48.1	82.68 ± 11.7	471.2 ± 126.9^$^
	POST_5MIN_	209.8 ± 58.1^$^	51.9 ± 42.8	81.69 ± 12.8	446.4 ± 105.9^$^
	POST_10MIN_	216.1 ± 53.8^$^	55.2 ± 52.4	79.97 ± 16.5	450.6 ± 84.9^$^

MVC_*K*_ – maximal voluntary contraction; PRE_*FATIGUE*_ – moment before the exercise application; POST_*FATIGUE*_ – moment at the end of the exercise; POST_5__*MIN*_ – 5th minute after the end of exercise; POST_10__*MIN*_ – 10th minute after the end of exercise; PerFat – Peripheral Fatigue Exercise; CenFat – Central Fatigue Exercise; Mom. – Moment; * - Significant difference with PRE_*FATIGUE*_ (*p* < 0.05); $ - Significant difference with PRE_*FATIGUE*_ (*p* < 0.01).

After both CenFat and PerFat, statistical differences were found for TP_E_ and several TS_E_ between PRE_FATIGUE_, POST_FATIGUE_, and both recovery parameters (*p* < 0.05). For TP_E_, effect sizes of the significant differences ranged between 0.853 and 0.453 after CenFat and between 1 and 0.965 after PerFat. Statistical differences were also observed for TS_E_ between POST_5__MIN_ and POST_10__MIN_ (*p* < 0.05) (Table 2).

**TABLE 2:** Neuromuscular parameters comparison of the elbow extensor muscles between the moments.

**Exercise**	**Mom.**	**TS_*E*_ 10%**	**TS_*E*_ 20%**	**TS_*E*_ 30%**	**TS_*E*_ 40%**	**TS_*E*_ 50%**	**TP_*E*_**
PerFat	PRE_*FATIGUE*_	2.4 ± 1.3	2.1 ± 1.1	1.8 ± 1.2	1.2 ± 0.7	0.8 ± 0.6	2.8 ± 1.0
	POST_*FATIGUE*_	1.1 ± 0.6*	0.8 ± 0.5*	0.5 ± 0.4*	0.4 ± 0.2*	0.3 ± 0.2*	1.2 ± 0.6*
	POST_5MIN_	1.4 ± 0.8*^#^	1.0 ± 0.6*	0.7 ± 0.5*	0.6 ± 0.5*	0.5 ± 0.6*	1.9 ± 0.9*^#^
	POST_10MIN_	1.2 ± 0.7*^&^	0.8 ± 0.6*	0.8 ± 0.6*	0.6 ± 0.4*	0.4 ± 0.3*	1.8 ± 0.9*^#^
CenFat	PRE_*FATIGUE*_	2.4 ± 1.0	2.1 ± 0.9	1.7 ± 0.8*	1.3 ± 0.6	0.8 ± 0.4	2.8 ± 1.1
	POST_*FATIGUE*_	1.6 ± 0.8*	1.3 ± 0.7*	1.0 ± 0.5*	0.7 ± 0.5*	0.5 ± 0.4*	2.2 ± 0.9*
	POST_5MIN_	2.0 ± 1.0*^#^	1.6 ± 0.9*^#^	1.3 ± 0.7*^#^	0.9 ± 0.5*	0.6 ± 0.3*	2.5 ± 1.0*^#^
	POST_10MIN_	2.1 ± 0.9*^&^	1.8 ± 0.9*^&^	1.5 ± 0.6*^&^	1.1 ± 0.6*^&^	0.7 ± 0.3*	2.4 ± 1.0*

*Parameters presented in N. PRE_*FATIGUE*_ – moment before the exercise application; POST_*FATIGUE*_ – moment at the end of the exercise; POST_5__*MIN*_ – 5th minute after the end of exercise; POST_10__*MIN*_ – 10th minute after the end of exercise; PerFat – Peripheral Fatigue Exercise; CenFat – Central Fatigue Exercise; Mom. – Moment; TS_*E*_ – Twitch Superimposed; TP_*E*_ – Twitch Potentiated; ^∗^ - Statistical difference with PRE_*FATIGUE*_; ^#^ - Statistical difference with POST_*FATIGUE*_; ^&^ - Statistical difference with POST_5__*MIN*_; ^$^ - Statistical difference with POST_10__*MIN*_ (Significances fixed at *p* < 0.05).*

### Differences Between Exercises

For lower limbs, it was found that TP_K_ values in the POST_FATIGUE_, POST_5__MIN,_ POST_10__MIN_ were lower after CenFat than PerFat (effect size: 0.884, 0.667, and 0.657, respectively; *p* < 0.01). MVC_K_ showed significant differences on POST_FATIGUE_, POST_5__MIN_, and POST_10__MIN_ (effect size: 0.379, 0.475, and 0.435 respectively; *p* < 0.05) (Table 3).

**TABLE 3:** Differences between exercises performed.

**Exercise**	**Mom.**	**TP_*K*_ (N)**	**TS_*K*_ (N)**	**VA_*K*_ (%)**	**MVC_*K*_ (N)**
PRE_*FATIGUE*_	PerFat	254 ± 63.6	55.5 ± 38.7	81.9 ± 9.3	597 ± 104.9
	CenFat	262 ± 58.1	62.9 ± 50.6	81.5 ± 11.9	546 ± 123.3
POST_*FATIGUE*_	PerFat	263 ± 65.6	62.6 ± 50.6	81.2 ± 16.5	562 ± 139.7
	CenFat	207 ± 44.9*	48.5 ± 48.1	82.7 ± 11.7	471 ± 126.9*
POST_5MIN_	PerFat	253 ± 65.9	59.9 ± 40.5	80.9 ± 10.7	544 ± 119.5
	CenFat	210 ± 58.1*	51.9 ± 42.8	81.7 ± 12.8	446 ± 105.9*
POST_10MIN_	PerFat	254 ± 65.0	63.4 ± 48.5	80.2 ± 11.2	539 ± 127.0
	CenFat	216 ± 53.8*	55.2 ± 52.4	80 ± 16.5	451 ± 84.9*

*Parameters obtained through muscle stimulation performed on knee extensor muscles. PRE_*FATIGUE*_ – moment before the exercise application; POST_*FATIGUE*_ – moment at the end of the exercise; POST_5__*MIN*_ – 5th minute after the end of exercise; POST_10__*MIN*_ – 10th minute after the end of exercise; PerFat – Peripheral Fatigue Exercise; CenFat – Central Fatigue Exercise; Mom. – Moment; TS_*K*_ – Twitch Superimposed; TP_*K*_ – Twitch Potentiated; VA_*K*_ – Voluntary activation; MVC_*K*_ – maximal voluntary contraction; ^∗^ - Statistical difference between exercises (*p* < 0.01).*

In upper limbs in the PerFat showed lower TS_E_ than CenFat on: POST_FATIGUE_ for TS_E_ on 10, 20, 30, 40, and 50% of the MVC_E_ (effect size: 0.674, 0.705, 0.700, 0.614, and 0.509 respectively; *p* < 0.05); POST_5__MIN_ for TS_E_ on 10, 20, and 30% of the MVC_E_ (effect size: 0.686, 0.733, and 0.752, respectively and 0.435 respectively; *p* < 0.05) and; POST_10__MIN_ for TS_E_ on 10, 20, 30, 40, and 50% of the MVC_E_ (effect size: 0.962, 0.971, 0.943, 0.781, and 0.789, respectively; *p* < 0.05). PerFat also showed lower TP_E_ than CenFat on POST_FATIGUE_, POST_5__MIN_, and POST_10__MIN_ (effect size: 0.987, 0.743, and 0.684; *p* < 0.01) (Table 4).

**TABLE 4:** Neuromuscular parameters comparison referring to the elbow extensor muscles by the exercises performed.

**Exercise**	**Mom.**	**TS_*E*_ 10%**	**TS_*E*_ 20%**	**TS_*E*_ 30%**	**TS_*E*_ 40%**	**TS_*E*_ 50%**	**TP_*E*_**
PRE_*FATIGUE*_	PerFat	2.41.3	2.11.1	1.81.2	1.20.7	0.80.6	2.81.0
	CenFat	2.41.0	2.10.9	1.70.8	1.30.6	0.80.4	2.81.1
POST_*FATIGUE*_	PerFat	1.10.6*	0.80.5*	0.50.4*	0.40.2*	0.30.2*	1.20.6*
	CenFat	1.60.8	1.30.7	10.5	0.70.5	0.50.4	2.20.9
POST_5MIN_	PerFat	1.40.8*	10.6*	0.70.5*	0.60.5	0.50.6	1.90.9*
	CenFat	21.0	1.60.9	1.30.7	0.90.5	0.60.3	2.51.0
POST_10MIN_	PerFat	1.20.7*	0.80.6*	0.80.6*	0.60.4*	0.40.3*	1.80.9*
	CenFat	2.10.9	1.80.9	1.50.6	1.10.6	0.70.3	2.41.0

*Parameters presented in N. PRE_*FATIGUE*_ – moment before the exercise application; POST_*FATIGUE*_ – moment at the end of the exercise; POST_5__*MIN*_ – 5th minute after the end of exercise; POST_10__*MIN*_ – 10th minute after the end of exercise; PerFat – Peripheral Fatigue Exercise; CenFat – Central Fatigue Exercise; Mom. – Moment; TS_*E*_ – Twitch Superimposed; TP_*E*_ – Twitch Potentiated; ^∗^ - Statistical difference between the exercises (*p* < 0.05).*

### Neuromuscular Coefficients

After CenFat, y-intercept on POST_FATIGUE_ was lower than PRE_FATIGUE_ (effect size: 0.884; *p* < 0.01) and higher than POST_10__MIN_ (effect size: 0.667; *p* < 0.01). After PerFat, y-intercept on PRE_FATIGUE_ was higher than POST_FATIGUE_, POST_5__MIN_, and POST_10__MIN_ (effect size: 0.853, 0.629, and 0.568, respectively; *p* < 0.05). After PerFat, slope on PRE_FATIGUE_ was higher than POST_FATIGUE_ and POST_5__MIN_ (effect size: 0.905 and 0.724 respectively; *p* < 0.05). After PerFat *R*^2^ on PRE_FATIGUE_ was higher than POST_FATIGUE_, POST_5__MIN_, and POST_10__MIN_ (effect size: 0.547, 0.533, and 0.633, respectively; *p* < 0.05) (Table 5).

**TABLE 5:** Mean values ± standard deviation of linear (y-intercept), angular (slope) and determination coefficients (*R*^2^).

**Exercise**	**Mom.**	**y-Intercept**	**Slope**	***R*^2^**
PerFat	PRE_*FATIGUE*_	69.89 ± 11.52	−29.69 ± 17.18	0.91 ± 0.08
	POST_*FATIGUE*_	55.95 ± 7.64*	−53.47 ± 35.77*	0.80 ± 0.21*
	POST_5MIN_	56.75 ± 12.53*	−51.93 ± 51.38*	0.74 ± 0.31*
	POST_10MIN_	58.85 ± 17.27	−42.60 ± 37.48*	0.66 ± 0.34*
CenFat	PRE_*FATIGUE*_	74.32 ± 18.94	−31.41 ± 14.98	0.94 ± 0.08
	POST_*FATIGUE*_	57.94 ± 22.29*	−26.59 ± 37.83	0.86 ± 0.17
	POST_5MIN_	66.32 ± 18.10	−35.86 ± 23.70	0.82 ± 0.19
	POST_10MIN_	68.34 ± 12.18^#^	−32.34 ± 17.06	0.87 ± 0.13

*Comparison between the moments for both exercises involving neuromuscular fatigue. All results are demonstrated by arbitrary units (AU). PRE_*FATIGUE*_ – moment before the exercise application; POST_*FATIGUE*_ – moment at the end of the exercise; POST_5MIN_ – 5th minute after the end of exercise; POST_10MIN_ –10th minute after the end of exercise; PerFat – Peripheral Fatigue Exercise; CenFat – Central Fatigue Exercise; Mom. – Moment; ^∗^ - significant difference between PRE_*FATIGUE*_ (*p* < 0.05); ^#^ - significant difference between POST_*FATIGUE*_ (*p* < 0.05).*

**FIGURE 3:**
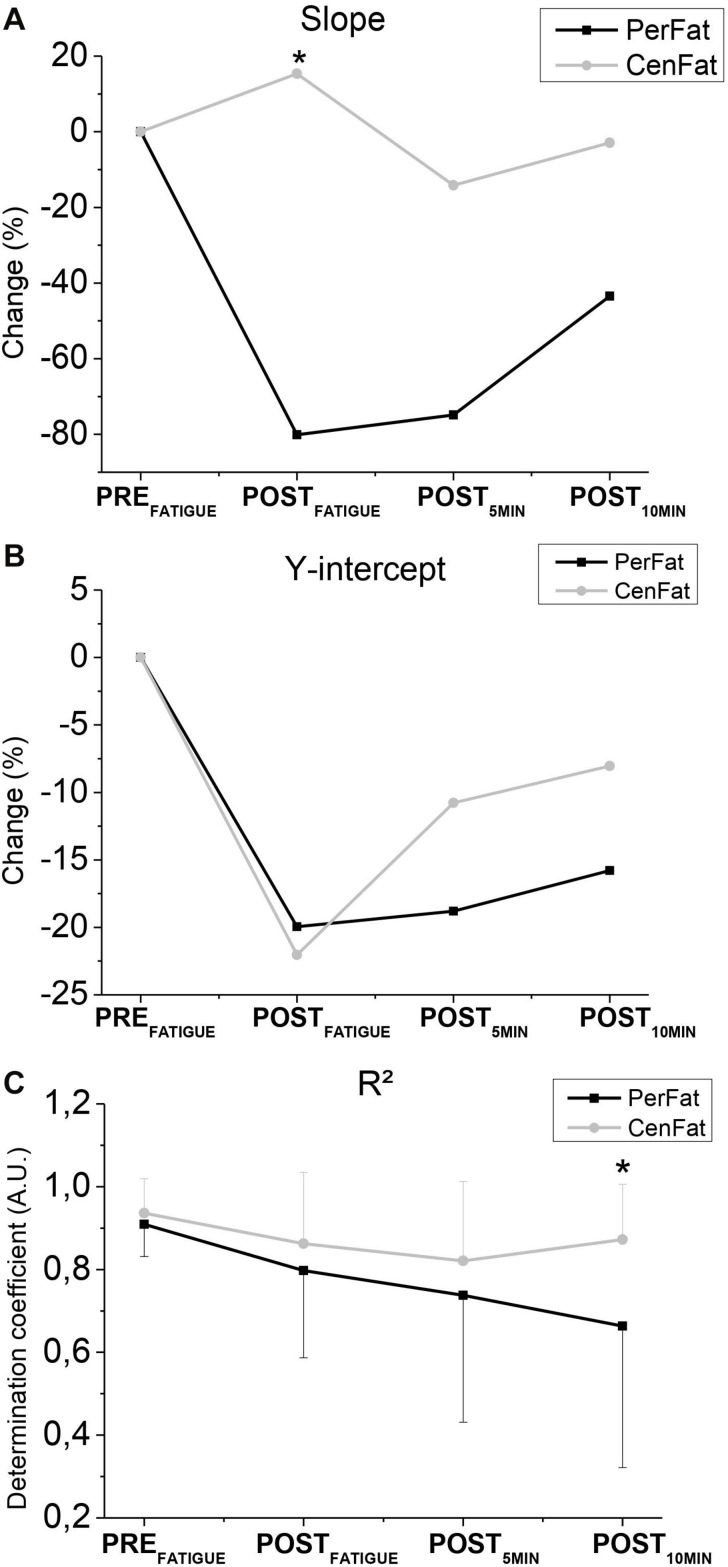
Representation of changes in slope **(A)**, y-intercept **(B)**, and *R*^2^ **(C)**. PerFat. – Exercise for induce peripheral fatigue; CenFat – Exercise for induce central fatigue; * - Statistical difference between the exercises (*p* < 0.05).

Slope was lower after PerFat than CenFat on POST_FATIGUE_ (effect size: 0.600; *p* < 0.05) (Figure 3A). *R*^2^ was higher after PerFat than CentFat on POST_10__MIN_ (effect size: 0.632; *p* < 0.05) (Figure 3C).

### Reliability Test

The reliability tests indicated good ICC indexes for TS_E_ at 10% (0.84) and 20% of MVC (0.82), TP_E_ (0.83) and TP_K_ (0.82). Moderate indices for TS_E_ were also found at 30% (0.74), Slope (0.50), *R*^2^ (0.62), MVC_K_ (0.59), TS_K_ (0.72), and AV_K_ (0.67). All reproducibility data are shown in Table 6.

**TABLE 6:** Reliability of the lower and upper limbs data showed by: interclass correlation coefficient (ICC), typical error of the measure (TE), coefficient of variation (CV), standard error of the measure (SEM), and minimal difference needed to be considered real (MD).

**Parameter**	**ICC (U.A.)**	**TE (U.A.)**	**CV (%)**	**SEM (U.A.)**	**MD (U.A.)**
TS_*E*_ on 10%	0,84^#^	0,57	11,78	0,33	0,90
TS_*E*_ on 20%	0,82^#^	0,49	11,61	0,29	0,81
TS_*E*_ on 30%	0,74*	0,50	14,63	0,36	1,00
TS_*E*_ on 40%	0,44	0,58	23,37	0,61	1,70
TS_*E*_ on 50%	0,16	0,57	38,03	0,74	2,06
TP_*E*_	0,83^#^	0,66	11,75	0,38	1,07
y-Intercept	−0,07	23,75	17,59	34,67	96,11
Slope	0,50*	11,57	−21,18	11,54	31,99
*R*^2^	0,62*	0,17	9,57	0,15	0,40
MVC_*K*_	0,59*	19,37	18,50	17,54	48,63
TP_*K*_	0,82^#^	2,47	4,69	1,46	4,06
TS_*K*_	0,72*	2,45	20,32	1,83	5,07
AV_*K*_	0,67*	5,75	3,52	4,64	12,87

*ICC and TC are presented in arbitrary units, CV in percentage and SEM and MD has the same units as the measurement evaluated. ICC: interclass correlation coefficient; TE: typical error of the measure; CV: coefficient of variation; SEM: standard error of the measure; MD: minimal difference needed to be considered real; MVC: maximal voluntary contraction; TS_*E*_: twitch superimposed on elbow extensor musculature; MVC_*K*_: maximal voluntary force of knee extensor musculature; TS_*K*_: twitch superimposed on knee extensor musculature; TP_*E*_: twitch potentiated on elbow extensor musculature; TP_*K*_: twitch potentiated on elbow extensor musculature; VA_*K*_: voluntary activation on knee extensor musculature; ^∗^ moderate index; # good index.*

## Discussion

The objective of the present study was to test a specific protocol for analyses of neuromuscular fatigue in elbow extensor muscles. Our main data show that the proposed protocol was able to verify changes in neuromuscular parameters using stimulation in submaximal contractions in the elbow extensor muscles.

The linear relationship between maintained submaximal force (10, 20, 30, 40, and 50% of MVC_E_) and the respective TS_E_ presented was able to find differences between the two efforts (Figure 3). However, it is not possible to confirm if this analysis method is able to discriminate central and peripheral fatigue since the exercise involving central fatigue did not present a significant central parameter (TS_K_) change (from 62.9 ± 50.6 N on PRE_FATIGUE_ to 48.5 ± 48.1 N on POST_FATIGUE_).

The results of the neuromuscular fatigue evaluation for the lower limbs was important to confirm the central fatigue absence for both protocols, considering that both exercises did not promote TSK increase (Table 1) (Allen et al., 1995; Gandevia, 2001).

When comparing the different moments, no statistical differences were found for TSK and VAK between PerFat and CenFat, contrasting that the stress experienced by both protocols was not able to weaken the central pathway considerably (Table 1). The MVC_K_ on lower limbs after PerFat and CenFat on knee extensor musculature can usually be associated with central fatigue (Gandevia, 2001) however, in the present study this was not accompanied by a TSK increase, indicating that the strength loss was provided in another way, possibly peripheral (Cè et al., 2020).

Although TP_K_ remained statistically unchanged after the PerFat, as expected, this information shows that exercise for upper limbs (PerFat) did not result in peripheral fatigue for lower limbs. However, a substantial decrease of these parameters was observed after CenFat, confirming peripheral fatigue manifestation (from 262.3 ± 58.1 N on PRE_FATIGUE_ to 206.7 ± 44.9 N on POST_FATIGUE_). Finally, it is important to point out that one of the exercises used (CenFat) was a limitation of the study because it did not cause central stress by changing TS_K_ (Gandevia, 2001).

The choice of the CenFat exercise model was based on a maximal strength training model proposed for knee extension that presented fatigue (Peltonen et al., 2013). In view of the limitation regarding gym equipment, we opted to use the training model in another exercise (leg extension) that also presented neuromuscular fatigue (without confirmation of central fatigue by twitch interpolation technique) (Linnamo et al., 1997). In theory, the use of more muscle groups guaranteed the promotion of central fatigue (Gandevia, 2001). This same exercise model made for CenFat (15 sets of a maximum repetition) has already been analyzed in another study that also showed peripheral fatigue. However, given its methodological limitations, it was not able to quantify the existence of central fatigue, despite highlighting the possible trend (Walker et al., 2012).

Analyzing the upper limb parameters, a reduction in TP_E_ was found after both exercises (Table 2). However, this reduction was more visible after PerFat when compared to CenFat (PerFat shows 1.2 ± 0.6 N and CenFat shows 2.2 ± 0.9 N at the POST_FATIGUE_ moment). These results suggest that both exercises provided some disturbance of the extensor elbow muscles (Allen et al., 1995; Gandevia, 2001). This situation was not expected after exercise involving central fatigue (CenFat).

The study of Bilodeau (2006) shows that the reduction in pulse applied to the relaxed musculature coincided with a muscle activity reduction (by surface electromyography) and VA_K_, thus the author concluded that there was central fatigue. That is, the author shows that TP_E_ responded to central fatigue. Despite the Bilodeau (2006) study highlighting the tendency to central fatigue that the triceps brachii possesses, in the present study none of the exercises caused central fatigue, considering TS_K_ (Gandevia, 2001) (Table 1).

It is possible that the TP_E_ reduction after the CenFat results from the participant’s handgrip of the leg extensor equipment (Leg-Press 45°), which contained support loops so that the participant could hold with their hands during the leg extension. However, further investigations should be made regarding this parameter’s representativeness for the neuromuscular protocol involving stimulation in the elbow extensor musculature.

Although both exercises showed TS_E_ reduction in all submaximal contraction intensities (Table 2), recovery of these parameters (POST_5__MIN_ and POST_10__MIN_) was different after each exercise. It was evidenced that, after PerFat, most of the TS_E_ (at each submaximal contraction intensity respectively) were similar, exhibiting a plateau behavior without signs of recovery, unlike the results after the CenFat, which showed signs of recovery (Table 2). Besides the difference between behaviors, it was also evidenced that the PerFat led to a more persistent stress when compared to the CenFat for most of the TS_E_ (Table 4).

The behavior resulting from the upper limb neuromuscular evaluation protocol, regardless of the participant’s neuromuscular state, was illustrated by an inverse relationship between the force evoked during the stimulation and the submaximal voluntary contraction rate performed, a scenario also reported by Temesi et al. (2014). Further studies (Becker and Awiszus, 2001; Cheng and Rice, 2010; Temesi et al., 2014) used several TS_K_ from different submaximal intensities of the knee extensor musculature to estimate the TP_K_. In addition, the report of evoked forceps linearity for different submaximal contractions has also been reported for biceps brachii (De Serres and Enoka, 1998; Todd et al., 2004; Cadigan et al., 2017).

As previously reported, in addition to identifying changes in neuromuscular parameters, the evaluation protocol suggested by the present study also showed linearity between the TS_E_ parameters (PerFat shows 0.91 ± 0.08 AU and CenFat shows 0.94 ± 0.08 AU in the PRE_FATIGUE_). Thus, the linear mathematical method used by Temesi et al. (2014) may be used safely and the resulting products can be studied as possible variables capable of identifying fatigue states. Despite the evidence regarding the importance of the curvelinear method (Behm et al., 1996; Shield and Zhou, 2004), there are no reports regarding the linearity for the triceps brachii. However, for the biceps brachii there are reports that both methods have similar validity (De Serres and Enoka, 1998).

The inherent “co-contraction phenomenon” (simultaneous contraction of the agonist and antagonist musculature) of the brachial musculature during stimulation is treated as an abnormality, and several authors describe this situation as limiting for neuromuscular analysis (Awiszus et al., 1997; Burke and Gandevia, 1998; Bilodeau, 2006; Cheng and Rice, 2010). However, this study considers that the existence of co-contraction (already proven in pilot and preliminary studies made by our group) should be studied as a natural state for certain muscular regions such as elbow extensor musculature, considering the impracticability of analyzing neuromuscular fatigue in isolation for specific muscles, since this type of analysis is only possible with dissection (*in vitro*) methods. In view of the possible underestimation of the evoked force, we used stimulation intensity that most evoked force (during the PET) and not necessarily the maximum intensity or near to the pain threshold of the subjects (Place and Millet, 2020).

Upon these considerations, the linear method used assumes the state of co-contraction as a natural state of the elbow extensor musculature. The linear method utilized by the present study shows the possible recovery tendency after the CenFat by the y-intercept parameter, whereas after the PerFat this parameter does not show recovery signs (Table 5).

It is important to note the contrary response of slope after both exercises (Figure 3). While PerFat induced a sudden reduction of slope, CenFat linear parameters obtained a discrete increase, contrasting different responses by the angulation of the linear method used. Despite the changes in angulation, the straight-line compliance was preserved since *R*^2^ values remained high, indicating low dispersion of the points for line formation.

The slope reduction after the PerFat may contrast three situations: reduction by the extremity that includes the “highest” stimulation values (TP_E_ and TS_E_ at 10% of MVC_E_); increase by the extremity comprising “lowest” stimulation values (TS_E_ at 40 and 50% of MVC_E_); and the set of both alterations mentioned.

Hypothetically, the mean decrease in slope can illustrate only the peripheral fatigue establishment, since TS_K_ did not show a significant change after PerFat (from 55.5 ± 38.7 N to 62.6 ± 50.6 N), a state that, theoretically, would not significantly change a possible TS_E_ at 100% of the MVC_E_ in the elbow extensor muscles (if we extend the linearity for higher contractions) (Gandevia, 2001). Furthermore, the TP_E_ sudden reduction after PerFat (to 2.8 ± 1.0 N from 1.2 ± 0.6 N) should be considered. This reduction is greater when compared after CenFat (PerFat shows 1.2 ± 0.6 N and CenFat shows 2.2 ± 0.9 N in the POST_FATIGUE_ moment – Table 4).

Although central fatigue was not observed, minimal central pathway stress influencing one-line extremity (referring to the TS_E_ at 40 and 50% of MVC_E_) accompanied by a reduction less accentuated by TP_E_ (Table 2), would be able to increase the slope in a discrete way. However, it is not possible to affirm that this variable increase is consistent with central fatigue since this scenario has not been significantly established (Table 1).

Although both exercises promote linear regression “disorder,” at POST_10__MIN_ *R*^2^ values are lower after PerFat, showing a more persistent disarray compared to the results after CenFat (Figure 3C). Linear regression may not be the best way to describe the fatigue recovery behavior (mainly peripheral), faced with the *R*^2^ decrease (Figure 3C).

Given the results found, it was evidenced that both protocols generated peripheral fatigue in the upper limbs (Table 2) contributing to a change in linear parameters (Figure 3). Further studies are needed to reveal whether these changes after CenFat are the result of peripheral fatigue provided by the equipment used or sensitivity to central path disturbances due to stress in a non-local region (Gandevia, 2001; Bilodeau, 2006; Halperin et al., 2015).

Halperin et al. (2015) showed that neuromuscular evaluations in the lower limbs are more effective in detecting non-local fatigue than upper limbs evaluation protocols. In addition, there is also evidence that central fatigue resulting from training stress in the dominant arm does not affect the activation of the non-dominant arm (Halperin et al., 2014). This information may indicate that (i) the arms are immune to the effect of contralateral central fatigue or; (ii) the evaluation protocol used for upper limbs (stimulation on 100% of MVC and stimulation on relaxed musculature) needs to be revised, considering that the task involving upper limbs provided a reduction in voluntary activation in lower limbs.

Finally, this scenario contrasts that if central fatigue may affect upper and lower limbs differently, it may be contradictory to analyze both in the same way. In this case, our study is a small step toward the field of neuromuscular knowledge and further studies will be needed to investigate neuromuscular fatigue through submaximal stimulations applied to the triceps brachii.

We also emphasize that the method showed good reliability for some parameters (Table 6), however, due to the difficulty in identifying the values of force evoked in higher submaximal contractions (40 ∼ 50%), other parameters proved to be poorly reproducible. This situation that contrasts the presence of noise during signal acquisition has been reported in other studies (Behm et al., 1996; Shield and Zhou, 2004; Paillard et al., 2005). In this sense, the evaluation routine should be repeated by testing another muscle stimulation format (e.g., electrode size, electrode type, electrode position, type of current applied, limb position).

## Conclusion

It is possible to conclude that the protocol using stimulation for the elbow extensor muscles presented sensitivity to verify neuromuscular fatigue. However, considering that CenFat did not significantly promote central pathway stress, some conclusions are limited.

The products of the linear relationship between maintained submaximal force and TS_E_ response (applied at different rates of submaximal contraction) is an effective method to identify peripheral fatigue. However, its ability to discriminate the origin of fatigue is limited, since the CenFat did not promote significant changes in central pathway.

Finally, linear regression appears to be a good method to investigate neuromuscular fatigue, although other non-linear methods should also be tested before this protocol has its validity tested in the practical environment.

### Additional Information

The study was designed by MP and MSN; data were collected and analyzed by MSN and TBA; critical procedure support was provided by MP; data interpretation and manuscript preparation were undertaken by MSN, TBA and MP. All authors approved the final version of the paper. The authors declare that the research was conducted in the absence of any commercial or financial relationships that could be construed as a potential conflict of interest. The study was possible due to the São Paulo Research Foundation (FAPESP) incentive and funding support (2016/10029-4, 2016/12781-5 and 2015/24833-7). Special thanks to all researchers in the aquatic activity laboratory for systematic support throughout the study progression.

## Data Availability Statement

The raw data supporting the conclusions of this article will be made available by the authors, without undue reservation.

## Ethics Statement

The studies involving human participants were reviewed and approved by Ethics Committee of School of Physical Education and Sport of Ribeirão Preto (Process number: 97168618.4.0000.5659) and conducted in accordance with the Declaration of Helsinki. The patients/participants provided their written informed consent to participate in this study.

## Author Contributions

MP and MN designed the study. MN and TA collected and analyzed the data. MP support the procedure. All authors approved data interpretation and manuscript preparation and the final version of the manuscript.

## Conflict of Interest

The authors declare that the research was conducted in the absence of any commercial or financial relationships that could be construed as a potential conflict of interest.
